# Intra-aortic Balloon Counterpulsation for High-Risk Percutaneous Coronary Intervention: Defining Coronary Responders

**DOI:** 10.1007/s12265-019-09871-8

**Published:** 2019-03-15

**Authors:** Natalia Briceno, Kalpa De Silva, Matthew Ryan, Tiffany Patterson, Kevin O’Gallagher, Howard Ellis, Simone Rivolo, Jack Lee, Simon Redwood, Ajay M. Shah, Michael Marber, Divaka Perera

**Affiliations:** 1 School of Cardiovascular Medicine and Sciences, British Heart Foundation Centre of Excellence and National Institute for Health Research Biomedical Research Centre, St. Thomas’ Hospital Campus, King’s College London, London, SE1 7EH UK; 2 Bristol Heart Institute, University Hospitals Bristol and University of Bristol, Bristol, UK; 3 King’s College London British Heart Foundation Centre of Research Excellence, The James Black Centre, 125 Coldharbour Lane, London, UK; 4 Department of Biomedical Engineering, School of Biomedical Engineering and Imaging Sciences, King’s College London, London, UK

**Keywords:** Intra-aortic balloon counterpulsation, Wave intensity analysis, Coronary perfusion efficiency, Coronary responders, Myocardial supply/demand ratio

## Abstract

The effect of intra-aortic balloon counterpulsation (IABC) varies, and it is unknown whether this is due to a heterogeneous coronary physiological response. This study aimed to characterise the coronary and left ventricular (LV) effects of IABC and define responders in terms of their invasive physiology. Twenty-seven patients (LVEF 31 ± 9%) underwent coronary pressure and Doppler flow measurements in the target vessel and acquisition of LV pressure volume loops after IABC supported PCI, with and without IABC assistance. Through coronary wave intensity analysis, perfusion efficiency (PE) was calculated as the proportion of total wave energy comprised of accelerating waves, with responders defined as those with an increase in PE with IABC. The myocardial supply/demand ratio was defined as the ratio between coronary flow and LV pressure volume area (PVA). Responders (44.4%) were more likely to have undergone complex PCI (*p* = 0.03) with a higher pre-PCI disease burden (*p* = 0.02) and had lower unassisted mean arterial (87.4 ± 11.0 vs. 77.8 ± 11.6 mmHg, *p* = 0.04) and distal coronary pressures (88.0 ± 11.0 vs. 71.6 ± 12.4 mmHg, *p* < 0.001). There was no effect overall of IABC on the myocardial supply/demand ratio (*p* = 0.34). IABC has minimal effect on demand, but there is marked heterogeneity in the coronary response to IABC, with the greatest response observed in those patients with the most disordered autoregulation.

## Introduction

Intra-aortic balloon counterpulsation (IABC) is still the most commonly used percutaneous cardiac assist device, employed for a variety of different indications such as myocardial protection during high-risk percutaneous coronary intervention (PCI) and treatment of cardiogenic shock. Despite decades of observational data suggesting physiological benefit [1–4], randomised controlled trials (RCT) have failed to demonstrate clinical benefit with routine use of counterpulsation in different clinical settings such as high-risk PCI, ST elevation myocardial infarction and cardiogenic shock [5–7]. However, across all of these trials, a significant minority of patients in the control arms have become compromised during the course of PCI and required bailout IABC insertion. Despite the uncertainty introduced by patient crossover from the conservative arms of these trials, there has been a downgrading of the recommendations for use in international guidelines [8], with a gradual decline in usage noted in recently published registries [9]. However, IABC still remains to be the most widely available circulatory support device in cardiac catheterisation laboratories internationally, due to its familiarity, ease of insertion and low cost.

The concept of IABC response has emerged from several clinical studies and registries from as early as the late 70s, suggesting that there is a cohort of patients that have a greater haemodynamic response to balloon counterpulsation [10–13], which may contribute to the lack of benefit seen in published RCTs. All of these studies have described response in terms of broad systemic haemodynamics such as cardiac index, diastolic pressure augmentation and pulmonary capillary wedge pressure. To date, there has been no characterisation of response to IABC therapy in terms of coronary haemodynamics with pan cardiac cycle or wave intensity analysis (WIA) parameters. Coronary WIA through the simultaneous acquisition of distal coronary pressure and flow has allowed researchers to understand the intricate interaction between the myocardium and coronary circulation in various different pathological states [14–16] and is a tool that has been used to understand the coronary effects of IABC therapy [1].

The main aims of this study were to comprehensively investigate the effects of balloon counterpulsation on invasive coronary and left ventricular (LV) physiology in a cohort of patients undergoing high-risk PCI, to investigate the impact of counterpulsation on cardiac coronary coupling through coronary wave intensity analysis and to define responders using coronary physiology to further characterise the cohort of patients most likely to benefit from IABC therapy.

## Methods

### Patient Population

Patients scheduled to undergo high-risk PCI with intra-aortic balloon pump support at two tertiary cardiac centres were eligible for enrolment. Inclusion criteria were an LV ejection fraction (LVEF) < 40%, complex coronary anatomy (left main stem, multi-vessel disease, bifurcation disease, the presence of coronary calcification) and extensive coronary disease (British Cardiovascular Intervention Society (BCIS) jeopardy score (JS) ≥ 6 [17]). Exclusion criteria were severe peripheral vascular disease precluding insertion of the IABP, cardiogenic shock or peri-procedural haemodynamic instability.

### Catheter Laboratory Protocol

**Fig. 1:**
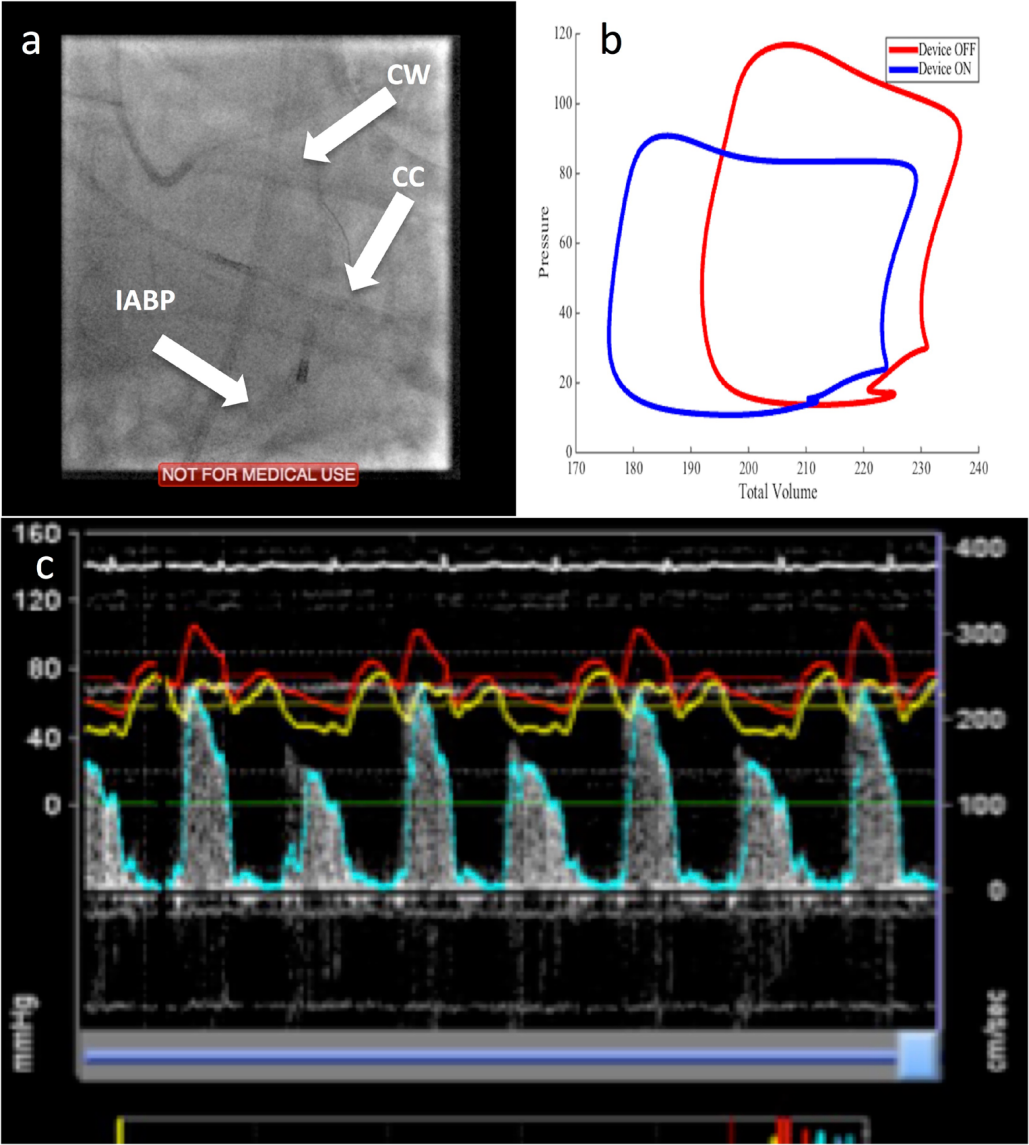
Study haemodynamic data acquisition. **a** Fluoroscopic placement of IABP catheter in the descending aorta, with ComboWire (CW) visualised in the left anterior descending artery and a PV loop catheter (CC) in the LV. **b** Representative PV loop obtained with and without IABC support. **c** Representative coronary data acquisition with the ComboWire and IABC support at 1:2 (red: aortic pressure, yellow: distal coronary pressure, blue: flow velocity (Doppler) trace)

All patients were preloaded with 300 mg of aspirin and 600 mg of clopidogrel. Three arterial sheaths were inserted under local anaesthetic: one in the radial artery (6F, for the guiding catheter) and one in each femoral artery (8F (for the LV conductance catheter where performed) and 9F (for the IABC device) sheaths respectively). Heparin was administered as standard for PCI procedures. An IABP catheter (Maquet, either 40 cc or MEGA 50 cc according to patient height) was inserted into the descending aorta distal to the subclavian artery origin and was activated with a counterpulsation ratio of 1:1. After PCI was completed, haemodynamic measurements were obtained in the aorta, target coronary artery and within the left ventricle. A 0.014″ dual pressure and Doppler flow guidewire (ComboWire XT, Philips) was inserted into the target coronary vessel through a fluid-filled guide catheter following pressure calibration. The coronary Doppler signal was optimised through manual adjustments of the wire tip within the vessel under fluoroscopic guidance. Following this in a subset of patients, an LV conductance catheter (CD Leycom, Zoetermeer, the Netherlands) was inserted into the LV along its longitudinal axis (see Fig. 1a). The catheter was connected to a signal conditioning and processing console, for real-time acquisition and display of pressure volume loops (see Fig. 1b). The conductance catheter technique has been previously described [18, 19]. This system allows the simultaneous acquisition of ventricular pressure and volume through a single pressure sensor and 12 evenly spaced electrodes on the pigtail catheter that measure time-varying segmental conductance. Volume correction was performed off-line post data acquisition, using 3D echocardiographic volumes obtained pre-PCI on day of the study protocol.

Following Doppler signal and LV pressure volume loop optimisation, measurements were obtained during 1:1 counterpulsation and with the balloon pump on standby, ensuring that heart rate, blood pressure and coronary average peak velocity had returned to baseline prior to a change in experimental condition (see Fig. 1c for representative coronary Doppler flow signals acquired during counterpulsation).

### Data Analysis

#### Coronary and Aortic Haemodynamic Data

Coronary data were sampled at 200 Hz and exported into dedicated software (Study Manager, Academic Medical Center, University of Amsterdam, Netherlands). A minimum of ten beats were selected for off-line analysis using custom-made software (Cardiac Waves, King’s College London, UK). Ensemble averages of at least five selected cardiac cycles were analysed with a Savitzky-Golay convolution method for smoothing of the mean aortic (*P*_*a*_) and coronary average peak velocity (APV) signals.

Pulse wave analysis was performed on central aortic pressure wave forms, with the calculation of the tension time index (TTI, area under the systolic portion of the aortic pressure wave form), the diastolic time index (DTI, area under the diastolic portion) and the Buckberg index (BI, index of myocardial supply and demand calculated as the ratio between DTI to TTI) [20]. Rate pressure product (RPP) was calculated by multiplying the heart rate by the systolic blood pressure (SBP). Diastolic augmentation was calculated as the augmented diastolic pressure during IABC minus unassisted diastolic aortic pressure. Systolic unloading was calculated as the unassisted SBP minus the SBP during IABC. Coronary microvascular resistance was calculated as mean *P*_*d*_ divided by APV.

Coronary wave intensity analysis was performed as previously described [15, 21]. In summary, net wave intensity (*δI*) was calculated from the time derivatives (*δt*) of the ensemble averaged distal coronary pressure (*P*_*d*_) and flow velocity (*U*), calculated as follows: *δI* = δ*P*_*d*_ / *δt* × *δU* / *δt*. The aortic-derived and microcirculatory-derived waves were separated assuming a blood density of 1050 kg m^−3^ and estimating a coronary wave speed using the sum of squares method [22]. The peak intensity of the four main waves was calculated (aortic-derived acceleratory forward compression wave (FCW), the microcirculatory-derived decceleratory backward compression wave (BCW), the aortic-derived decceleratory forward expansion wave (FEW) and the microcirculatory-derived backward expansion wave (BEW), considered to be the main contributor of coronary perfusion [15]). During IABC therapy, two further waves were identified as previously described, the IABP-FCW (acceleratory associated with balloon inflation) and the IABP-FEW (decceleratory wave) [1]. Perfusion efficiency (PE) was calculated as the percentage of accelerating waves of the total wave energy, using the area under the curve for each wave [16, 23]. For the calculation of PE during balloon counterpulsation, the IABP-forward compression wave (IABP-FCW) and IABP-forward expansion wave (IABP-FEW) were added to the total acceleratory and decceleratory wave energies respectively.

#### LV Haemodynamic Data

LV haemodynamic data was extracted and imported into custom-made software (Simplewires, King’s College London, UK) following the application of a 25-Hz filter for off-line analysis. At least ten consecutive cycles were selected from each condition, with the ensemble average of at least five beats within the sample used for data analysis. Left ventricular conductance volumes (EDV and ESV) were calculated at the maximum rate of pressure rise during systole (*dP*/*dT*_max_) and pressure decay during diastole (*dP*/*dT*_min_). Stroke volume was calculated as EDV minus ESV. LV stroke work was calculated as the area within the pressure volume loop. Pressure volume area (PVA), which is a measure of total mechanical energy generated by ventricular contraction, was calculated as the sum of stroke work and potential energy as previously described [24]. A myocardial supply/demand ratio was calculated as the ratio between coronary APV and PVA.

#### Classification of Coronary Responders

Coronary responders were defined as those patients that had an increase in coronary PE during IABC therapy. PCI was classed dichotomously as complex or not on the basis of lesion characteristics, contrast volume and use of adjunctive devices such as rotational atherectomy.

### Statistical Analysis

Statistical analysis was performed using IBM SPSS Version 24. Normality was assessed qualitatively through the review of histograms and quantitatively with the Shapiro-Wilk test. Normally distributed data is presented as mean ± SD, and non-normally distributed data is presented as median (interquartile range). Normally distributed data were compared using paired *t* tests, and non-normally distributed data were compared with the Mann-Whitney *U* test. Categorical data was compared with the chi-squared test. A *p* value of < 0.05 was considered significant. Correlation analysis was performed with the use of the Pearson correlation coefficient.

### Ethical Conduct and Approval Statement

All procedures followed were in accordance with the ethical standards of the responsible committee on human experimentation (UK National Health Service Research Ethics Service, Research Ethics Committee London Bridge, reference 11/H0804/10) and with the Helsinki Declaration of 1975, as revised in 2000. Informed written and verbal consent was obtained from all patients enrolled into the study.

## Results

### Patient and Procedural Characteristics

Thirty-six patients were enrolled, but nine were excluded due to haemodynamic instability during the procedure or vascular access difficulties. Twenty-seven patients (age 70 ± 9 years, 89% male, LVEF 31% ± 9%, JS 10 ± 2) underwent coronary assessment during device support. The last nine patients enrolled also underwent LV haemodynamic assessment, following successful approval of a substantial amendment to the local ethics committee. A 40-cc intra-aortic balloon pump catheter was used in 81% of cases and a 50-cc catheter in the others. Overall, there was no in-hospital mortality, and event rates were low.

### Effect of IABC on Systemic, Coronary and Left Ventricular Haemodynamics

IABC resulted in systolic unloading and diastolic augmentation (see Table 1), with a significant reduction in rate pressure product (7857.9 (6270.8, 10,270.23) vs. 6279.0 (5089.0, 8210.6), *p* = 0.001). There was a numerical increase in mean arterial pressure with IABC (*p* = 0.07). Balloon pump activation resulted in a significant reduction in LV end systolic pressure (109.5 ± 17.7 vs. 88.5 ± 16.5 mmHg, *p* < 0.001) and a reduction in LV end diastolic pressure (19.0 ± 10.3 vs. 15.6 ± 7.5 mmHg, *p* = 0.05); see Table 1. There was a significant reduction in afterload, as measured by arterial elastance (3.0 ± 1.4 vs. 2.1 ± 0.9 mmHg mL, *p* = 0.03). However, there was no difference in LV stroke work or PVA with balloon activation (*p* = 0.77 and *p* = 0.33 respectively), with no difference in the myocardial supply/demand ratio (0.0040 ± 0.0046 vs. 0.0049 ± 0.0043, *p* = 0.34).

**Table 1:** Aortic and left ventricular haemodynamic characteristics

	Unassisted	Assisted	*p* value
Aortic parameters			
HR (beats min^−1^)	73.5 ± 15.7	71.5 ± 12.9	0.39
SBP (mmHg)	114.4 ± 21.0	98 ± 26.8*	< 0.001
DBP (mmHg)	64.4 ± 10.1	127.8 ± 38.7*	< 0.001
Pa (mmHg)	83.1 ± 12.1	88.8 ± 23.4	0.07
RPP (mmHg bpm)	7857.9 (6270.8, 10,270.2)	6729.0 (5089.9, 8210.6)*	0.001
DTI	35.6 (30.0, 44.3)	49.7 ± 14.9*	< 0.001
ITI	33.7 (26.2, 36.6)	26.3 ± 9.3*	< 0.001
BI	1.2 (0.9, 1.4)	2.0 (1.6, 2.3)	< 0.001
LV haemodynamics			
EDP (mmHg)	19.0 ± 10.3	15.6 ± 7.5	0.05
ESP (mmHg)	109.5 ± 17.6	88.5 ± 16.5*	< 0.001
SV (mL)	40.9 (32.9, 46.8)	46.4 (29.4, 55.9)	0.09
*dP*/*dt*+ (mmHg s^−1^)	862.3 ± 90.2	788.8 ± 117.5*	0.03
*dP*/*dt*− (mmHg s^−1^)	− 873.9 ± 119.1	− 710.5 ± 110.0*	0.001
CO (L min^−1^)	3.0 (1.7, 3.4)	3.3 (2.1, 4.5)	0.14
SW (mmHg mL)	3676.4 (2683.5, 4565.5)	3562.4 (2292.2, 4144.8)	0.77
PVA	10,967.4 ± 7711.7	10,042.3 ± 6826.9	0.33
Arterial elastance (mmHg mL^−1^)	3.0 ± 1.4	2.1 ± 0.89	0.003

*p < 0.05 unassisted vs assisted

IABC resulted in an increase in distal coronary pressure (80.7 ± 14.0 vs. 84.9 ± 26.8 mmHg, *p* = 0.04), an increase in coronary flow (APV 27.2 ± 13.5 vs. 33.9 ± 13.6 cm s^−1^, *p* = 0.001) and a reduction in coronary microvascular resistance (2.9 (2.5, 4.6) vs. 2.7 (2.1, 3.4) mmHg cm s^−1^, *p* = 0.049); see Table 2.

**Table 2:** Coronary haemodynamic characteristics

	Unassisted	Assisted	*p* value
Coronary pan cardiac cycle indices			
Pd (mmHg)	80.7 ± 14.1	84.9 ± 18.2*	0.04
APV (cm s^−1^)	27.2 ± 13.5	33.9 ± 17.6*	0.001
MR (mmHg cm s^−1^)	2.9 (2.5, 4.6)	2.7 (2.1, 3.4)*	0.049
Coronary WIA (W m^−2^ s^−2^ × 10^5^)			
FCW	0.62 (0.37, 0.89)	0.67 (0.33, 1.57)	0.19
BCW	− 0.59 (− 0.78, − 0.24)	− 0.65 (− 0.98, − 0.29)	0.39
FEW	0.17 (0.04, 0.38)	0.12 (0.04, 0.28)	0.47
BEW	− 1.28 (− 1.81, − 0.93)	− 1.20 (− 3.10, − 0.81)	0.29
IABP-FCW	n/a	1.42 (0.77, 2.09)	n/a
Perfusion efficiency	79.0 (65.8, 86.4)	73.7 (69.2, 83.2)	0.77

*p < 0.05 unassisted vs assisted

**Fig. 2:**
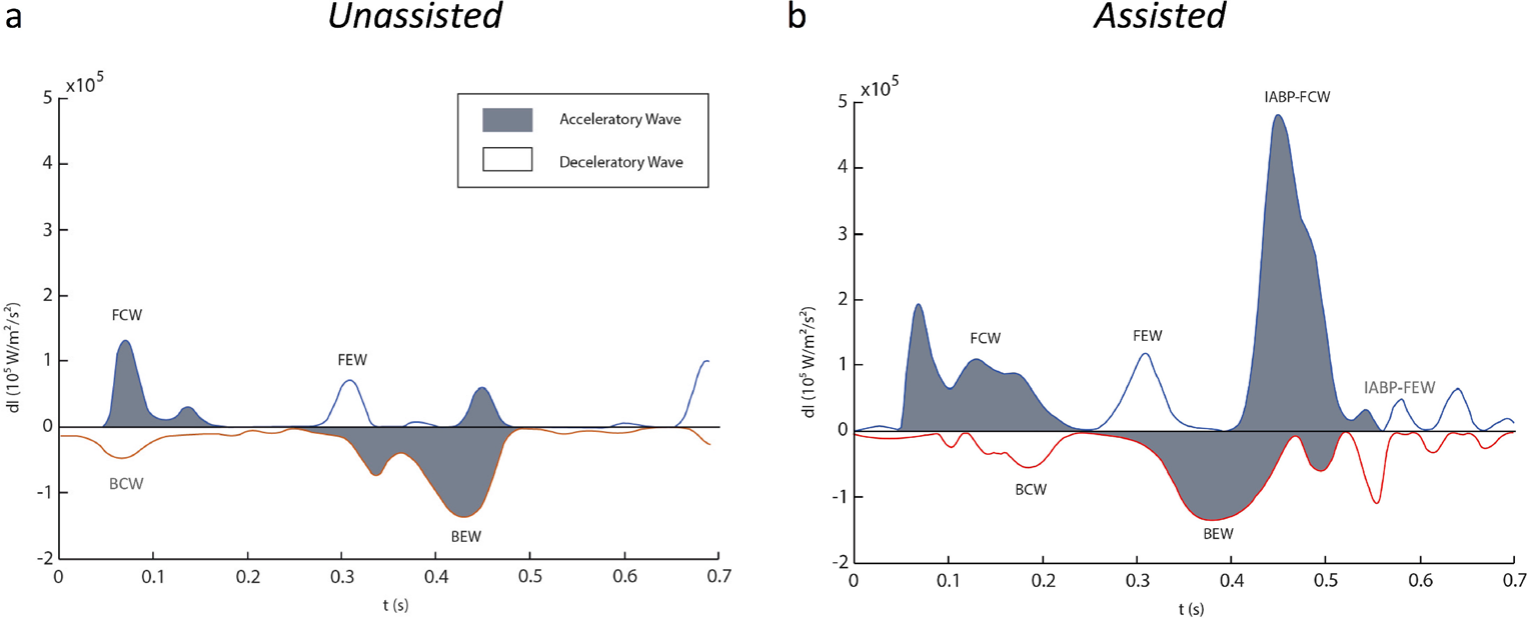
Representative WIA profile during unassisted (**a**) and assisted (**b**) conditions. The four main coronary waves are depicted (FCW forward compression, BCW backward compression wave, FEW forward expansion wave, BEW backward expansion wave). In **b**, the two IABP balloon inflation and deflation related waves are depicted (IABP-FCW IABP-forward compression wave, IABP-FEW IABP-forward expansion wave)

**Fig. 3:**
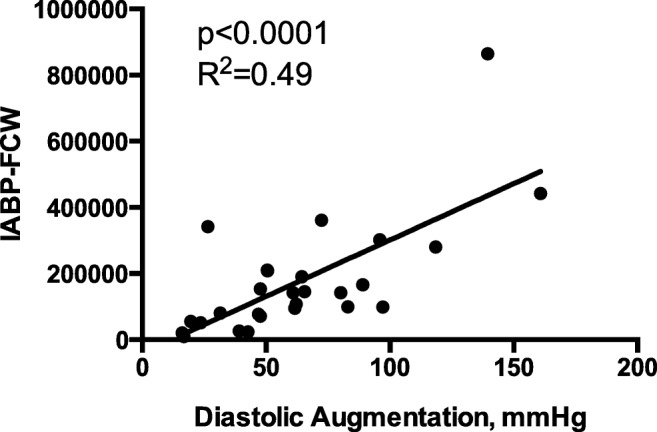
IABP relationship with diastolic augmentation. During IABC, the IABP-FCW is linearly correlated with the degree of diastolic aortic pressure augmentation

During unassisted conditions, the four main previously described coronary wave energies were identified in all patients. During counterpulsation, an acceleratory IABP-FCW was identified that relates to balloon inflation and a decceleratory IABP-FEW wave was identified in all patients; see Fig. 2. The IABP-FCW correlated with the degree of diastolic augmentation (*p* < 0.001, *R*^2^ = 0.49); see Fig. 3.

### Defining Coronary Haemodynamic Responders

There was no significant difference in peak wave intensity for each of the four waves during unassisted and assisted conditions (see Table 2 for mean peak wave energies). IABC therapy had a variable impact on perfusion efficiency, which ranged between − 36.2% and 100.3% across the cohort. Of patients, 44.4% had an increase in perfusion efficiency with IABC and were classed as coronary responders. There was no significant difference in demographics between non-responders and responders (see Table 3). Coronary responders were more likely to have a greater extent of coronary disease (BCIS JS 9.1 ± 2.5 vs. 11 ± 1.3, *p* = 0.02) and to have undergone more complex (*p* = 0.03) and multi-vessel PCI (*p* = 0.04).

**Table 3:** Patient and procedural characteristics

	Whole cohort *n* = 27	Non-responders *n* = 15	Responders *n* = 12
Age (years)	70 ± 9	70.9 ± 7.0	68.9 ± 10.9
Male	24 (89)	14 (93)	12 (86)
Hypertension	20 (74)	13 (87)	7 (58)
Diabetes	11 (41)	7 (47)	4 (33)
Hypercholesterolaemia	22 (81)	14 (93)	8 (67)
History of prior MI	13 (48)	7 (47)	7 (58)
LVEF (%)	31 ± 9	31.3 ± 8.4	30.3 (10.6)
BCIS-JS	10 ± 2	9.1 ± 2.5	11 ± 1.3^Λ^
Number of stents	1.9 ± 0.9	1.8 ± 1.0	2.1 ± 0.8
Multi-vessel PCI	8 (30)	3 (20)	7 (58)^Λ^
Rotational atherectomy	1 (4)	0 (0)	1 (8)
Procedural time (min)	106 ± 27	102.9 ± 22.3	109.8 ± 31.2
In-hospital mortality	0 (0)	0 (0)	0 (0)
Peri-procedural inotropes/vasopressors	0 (0)	0 (0)	0 (0)
Post PCI complications	2 (7)	1* (7)	1** (8)

Data are mean ± SD or *n* (%)

*No reflow post PCI

**Atrial fibrillation post procedure and cerebral embolus in the same patientc

^Λ^*p* < 0.05 non-responders versus responders

**Fig. 4:**
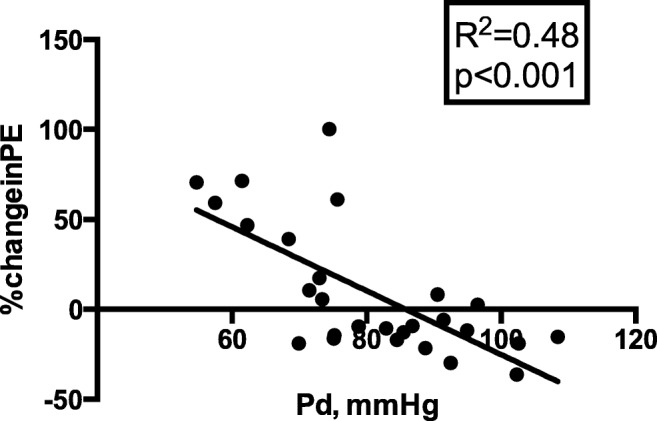
Relationship between distal coronary pressure during unassisted conditions and percentage change in perfusion efficiency with IABC therapy. The lower the distal coronary pressure, the greater the increment in perfusion efficiency with IABC

There was no significant difference in the degree of diastolic augmentation or systolic unloading (see Table 4). Responders had a lower distal coronary pressure (88.0 ± 11.0 vs. 71.6 ± 12.4 mmHg, *p* < 0.001) and mean aortic pressure (87.4 ± 11.0 vs. 77.8 ± 11.6 mmHg, *p* = 0.04) during unassisted conditions. There was no difference in unassisted microvascular resistance (3.19 (2.56, 4.51) vs. 2.51 (1.98, 5.08) mmHg cm s^−1^, *p* = 0.37) or coronary flow between groups (27.2 ± 11.0 vs. 27.2 ± 16.1 cm s^−1^, *p* = 1). The percentage change in PE negatively correlated with the distal coronary pressure (*R*^2^ = 0.48, *p* < 0.001, see Fig. 4) and diastolic blood pressure (*R*^2^ = 0.36, *p* < 0.001) during unassisted conditions.

**Table 4:** Coronary responder and non-responder haemodynamics

	Non-responders *n* = 15	Responders *n* = 12	*p* value
Aortic parameters			
Diastolic augmentation (mmHg*)	65.1 ± 39.0	61.2 ± 34.4	0.8
Systolic unloading (mmHg*)	12.7 ± 17.7	20.9 ± 12.0	0.19
Pa (mmHg)	87.4 ± 11.0	77.8 ± 11.6	0.04*
RPP (mmHg bpm)	88,978.7 ± 2809.8	7722.2 ± 1605.8	0.18
BI	1.1 ± 0.41	1.3 ± 0.3	0.18
Coronary pan cardiac cycle indices			
Pd (mmHg)	88.0 ± 11.0	71.6 ± 12.4	< 0.001*
APV (cm s^−1^)	27.2 ± 11.8	27.2 ± 16.1	1.00
MR (mmHg cm s^−1^)	3.2 (2.5, 4.5)	2.5 (2.0, 5.1)	0.9
Coronary WIA (W m^−2^ s^−2^ × 10^5^)			
FCW	0.74 ± 0.42	0.58 ± 0.46	0.35
BCW	− 0.49 ± 0.24	− 0.83 ± 0.64	0.07
FEW	0.12 ± 0.15	0.45 ± 0.37	< 0.001*
BEW	− 1.8 ± 1.2	− 1.3 ± 0.9	0.22
IABP-FCW*	1.4 ± 1.2	2.3 ± 2.3	0.21
Perfusion efficiency (%)		62.6 ± 16.7	< 0.001*
LV haemodynamics			
EDP (mmHg)	20.6 ± 13.5	17.8 ± 8.4	0.70
ESP (mmHg)	111.4 ± 13.9	108.0 ± 21.8	0.80
SV (mL)	42.0 ± 5.3	50.1 ± 44.8	0.70
SW (mmHg mL)	4084.2 ± 1015.9	4414.1 ± 4160.6	0.90
PVA (mmHg mL)	10,241.2 ± 6211.9	11,548.3 ± 9436.6	0.80
Arterial elastance (mmHg mL^−1^)	2.7 ± 0.3	3.3 ± 1.9	0.50

All data presented was obtained during unassisted conditions apart from those haemodynamic parameters annotaed with *

*p < 0.05 non-responders versus responders

In the nine patients with paired coronary and LV haemodynamic data, there were no differences between responders or non-responders in LV end systolic pressure, LVEDP, ventricular volumes or arterial elastance. There was no difference in LV stroke work (4084.2 ± 1015.9 vs. 4414.14160.6 mmHg mL, *p* = 0.9) or PVA (10,241.2 ± 6211.9 vs. 11,548.3 ± 9436.6 mmHg mL, *p* = 0.8). Numerically, there was a decline in PVA with IABC therapy in responders, which did not reach statistical significance (delta PVA 591.9 ± 2833.3 vs. − 2138.7 ± 2077.9 mmHg mL, *p* = 0.14).

## Discussion

The following are the main findings of this study:

1. The effects of IABC on supply are more marked than those on demand, with a neutral effect seen on the myocardial supply/demand ratio.
2. There is marked variability in coronary response to IABC as demonstrated by the range in change in perfusion efficiency, with less than half of the patients classed as responders.
3. Coronary responders were more likely to have a higher burden of coronary disease, to have undergone more complex PCI and to have lower unassisted aortic and distal coronary pressures following PCI.

Intra-aortic balloon pump counterpulsation has been the staple percutaneous haemodynamic support device for more than 40 years [25] and up until recently the only device available to the interventional cardiologist for haemodynamic support. We are currently in an era where increasing aged patients undergo PCI, and inevitably, they have a higher burden of co-morbidities, including prior AMI and impaired LV function. This poses a significant challenge, as it is known that the presence of LV dysfunction increases mortality following PCI [26]. Research has therefore been focused on strategies to reduce the ischaemic burden and subsequent complications, via a positive impact on the myocardial supply/demand ratio. Despite its ease of use, availability and extensive favourable physiology and registry data, IABC deployment is declining due to the publication of several neutral randomised trials that have all concluded that routine use of IABC therapy in various clinical settings does not alter outcomes [5–7]. However, there is a consistent subset of patients in the control arms of each of the studies that required bail out therapy, suggesting a group that may benefit. It is these patients that need to be identified, through the investigation of clinical characteristics and of predictors of response. There are clinical situations emerging where the use of IABC therapy may be beneficial. Van Nunen et al. studied a subgroup of patients from the CRISP-AMI trial, who had a large anterior MI and persistent ischaemia as defined by poor ST segment resolution [27]. In this group, IABC reduced 6-month mortality and there was a reduction in the composite end point. Despite the limitations of a small sample size and the fact that this study was a subgroup analysis of a larger clinical trial, the data is hypothesis generating and suggests a clinical cohort who may benefit.

The physiological effects of IABC have been extensively studied, both in animal models and clinical studies. These studies have confirmed the balloon pump’s effect on aortic haemodynamics, with diastolic augmentation and systolic unloading being its predominant effects. Previous studies on the effects of IABC on coronary flow have demonstrated that it is in those that have more deranged haemodynamics [3] or evidence of disabled autoregulation following administration of adenosine [1] that there is an observed augmentation in coronary flow with counterpulsation, eluding to a variability in haemodynamic response to IABP therapy between patients. In the current study, we demonstrated that IABC augments coronary flow without the administration of adenosine. The difference in our results compared with our group’s previous work may reflect the type of patients included. The LVEF on average in this study was 31%, whereas in our group’s previous work, the average EF was 34%. The patients enrolled in our study also had a higher BCIS jeopardy score, suggesting a group overall that have a larger amount of myocardium at risk and therefore at baseline may have had disordered autoregulation. This study has also begun to unravel the effects of IABC on coronary wave intensity analysis, with the observation that the degree of diastolic augmentation correlates with the magnitude of the IABP-FCW. It is important to note that in our study and in the prior studies discussed, coronary flow data was obtained following PCI, once the stenosis had been relieved. Previous studies have demonstrated no significant effect of IABC on distal coronary flow velocity in patients with critical coronary artery disease [2, 28], to suggest a differential effect of IABC therapy depending on the presence or absence of a stenosis. In the study by Kern et al. [2], it was only following PCI that an augmentation of coronary flow was observed with IABC. In this present study, we were unable to obtain coronary physiology data prior to PCI, and therefore, the physiological effects seen are with an open coronary artery free of significant disease.

In this study, we have demonstrated that the effects of counterpulsation are less pronounced on the left ventricle than those on the coronary circulation with no effects observed on stroke work or pressure volume area, with a resultant neutral effect of IABC on the myocardial supply and demand ratio. Previous animal [29, 30] and clinical studies [11] have not demonstrated consistent effects on stroke work and pressure volume area, but these studies present a variety of different heart failure models and different patient cohorts. Whether the current results explain why in particular patients in cardiogenic shock have not been shown to derive a clinical benefit (where there is a predominant myocardial issue), alongside its overall modest effect on cardiac output and mean arterial pressure, needs to be evaluated further. It may be that devices that off load the LV such as the Impella device may have more significant effects on reducing myocardial demand.

Previous studies have defined IABP responders as those patients who have a significant systemic haemodynamic effect from counterpulsation, whether it be the degree of augmentation of cardiac index, reduction of pulmonary capillary wedge pressure or effects on other haemodynamic indices such as left and right heart filling pressures. A clinical study of 76 patients undergoing IABP therapy with a 50-cc balloon pump defined responders as those that had a higher cardiac output after IABP insertion (*n* = 60) and those that did not (*n* = 16) [31]. They found that the best predictor of response was having a baseline cardiac index of 0.3 or less, but no difference in hospital mortality was observed between groups. An invasive LV pressure volume loop study in patients with advanced heart failure also tried to address the issue of response [11], defining response according to the degree of increment in cardiac index above the median for the group. They observed that overall, patients defined as responders had lower right-sided filling pressures and higher systemic vascular resistance. In this study, responders were defined on the basis of IABC effects on coronary haemodynamics, the first to date to define responders in this way. There was an observed large range of change in perfusion efficiency with IABC, which again is reflective of the clinical heterogeneity despite using broad diagnostic criteria to enrol these patients into the study. We found that responders had a higher BCIS jeopardy score and were more likely to have undergone complex multi-vessel PCI. We also found that responders had lower mean distal coronary and aortic pressures during basal conditions, with both demonstrating a negative correlation with percentage change in perfusion efficiency with IABC. These observations further add to the body of evidence emerging that it is in those patients that have worse coronary haemodynamics at the outset (with worse perfusion efficiency and exhausted autoregulation), a larger ischaemic burden, and that undergo more complex PCI are those that are more likely to respond physiologically to IABC therapy. There were no obvious differentiators in terms of LV haemodynamics, but this may be in part related to the small sample size of patients undergoing paired coronary and LV haemodynamic assessment. Further studies need to be performed that enrol patients with more narrowly defined physiological parameters to understand the effect of IABC on clinical outcomes.

### Limitations

This is a small physiological study and may be prone to type 1 error due to the small sample size. However, this is the largest invasive study to date on balloon pump coronary and LV physiology. As mentioned above, all measurements were obtained post PCI, and we therefore may not be able to translate these results to those patients with untreated severe coronary artery disease. Measurements were also taken in clinically stable patients, which is reflected by our low event rates. It is likely that the physiology of IABC is different in patients with haemodynamic instability or cardiogenic shock, again limiting the translatability of our observations The use of perfusion efficiency as a means to define responders is not clinically applicable, due to its invasive nature and reliance of good coronary Doppler signals to perform WIA; however, it has allowed us to begin to understand which patients may derive a greater benefit in terms of coronary haemodynamics.

## Conclusion

This is the largest invasive clinical study to date of balloon counterpulsation, reaffirming its significant effects on the coronary circulation, with an augmentation in coronary flow observed regardless of the autoregulatory state. The effects on LV haemodynamics are less pronounced. IABP responders in this cohort as defined by the change in perfusion efficiency were more likely to have disordered autoregulation with worse systemic haemodynamics, lower distal coronary pressure, and were more likely to have a higher ischaemic burden and to have undergone complex PCI.

### Clinical Implications

The use of IABC has been questioned, and use is on the decline. This study highlights the heterogeneous physiological response to IABC therapy despite using the same broad clinical criteria to select patients for enrolment, which may explain the lack of benefit seen in large randomised controlled trials. The findings that in those patients with the most deranged haemodynamics following PCI have a greater coronary response may aid the design of future clinical trials to establish patient cohorts that may derive benefit, to ultimately aid clinicians in selecting patients for IABC therapy.

## Abbreviations

IABC: Intra-aortic balloon counterpulsation
PCI: Percutaneous coronary intervention
RCT: Randomised controlled trial
LVEF: Left ventricular ejection fraction
APV: Average peak velocity
RPP: Rate pressure product
DTI: Diastolic time index
TTI: Tension time index
BI: Buckberg index
WIA: Wave intensity analysis
FCW: Forward compression wave
BEW: Backward expansion wave
BCW: Backward compression wave
FEW: Forward expansion wave
IABP-FCW: Intra-aortic balloon pump-forward compression wave
IABP-FEW: Intra-aortic balloon pump-forward expansion wave
PE: Perfusion efficiency
EDV: End diastolic volume
ESV: End systolic volume
PVA: Pressure volume area
